# Supplementary material for: In-depth characterization of denitrifier communities across different soil ecosystems in the tundra
Source: Environ Microbiome. 2022 Jun 11;17:30. doi: 10.1186/s40793-022-00424-2 (PMC9188126; doi:10.1186/s40793-022-00424-2)
Supplement: Supplementary file 2 — Additional file 2. Fig. S1. Physicochemical composition of tundra soils in Kilpisjärvi, northern Finland. Fig. S2. The microbial diversity of Kilpisjärvi soils as seen using a gene-centric approach. Fig. S3. Genome-resolved metagenomics of tundra soils. Fig. S4. Overview of the microbial diversity in Kilpisjärvi soils based on a genome-resolved approach. Fig. S5. Metabolic potential for denitrification in Stordalen Mire soils. Fig. S6. Phylogeny of a) nirK, b) nirS, c) norB, and d) nosZ sequences from metagenome-assembled genomes (MAGs) recovered from tundra soils in Kilpisjärvi, northern Finland. [file 40793_2022_424_MOESM2_ESM.docx]

*BMC Environmental Microbiome*

In-depth characterization of denitrifier communities across different soil ecosystems in the tundra

Igor S. Pessi^1,2^, Sirja Viitamäki^1^, Anna-Maria Virkkala^3,4^, Eeva Eronen-Rasimus^1,5^,
Tom O. Delmont^6^, Maija E. Marushchak^7,8^, Miska Luoto^4^, and Jenni Hultman^1,2,9,*^

^1^Department of Microbiology, University of Helsinki, Helsinki, Finland

^2^Helsinki Institute of Sustainability Science (HELSUS), Helsinki, Finland

^3^Woodwell Climate Research Center, Falmouth, MA, USA

^4^Department of Geosciences and Geography, University of Helsinki, Helsinki, Finland

^5^Marine Research Centre, Finnish Environment Institute (SYKE), Helsinki, Finland

^6^Génomique Métabolique, Genoscope, Institut François-Jacob, CEA, CNRS, Université d'Evry, Université Paris-Saclay, Evry, France

^7^Department of Biological and Environmental Science, University of Jyväskylä, Jyväskylä, Finland

^8^Department of Environmental and Biological Sciences, University of Eastern Finland, Kuopio, Finland

^9^Natural Resources Institute Finland (LUKE), Helsinki, Finland

^*^Corresponding author: jenni.hultman@helsinki.fi

# Additional file 2: Supplementary figures


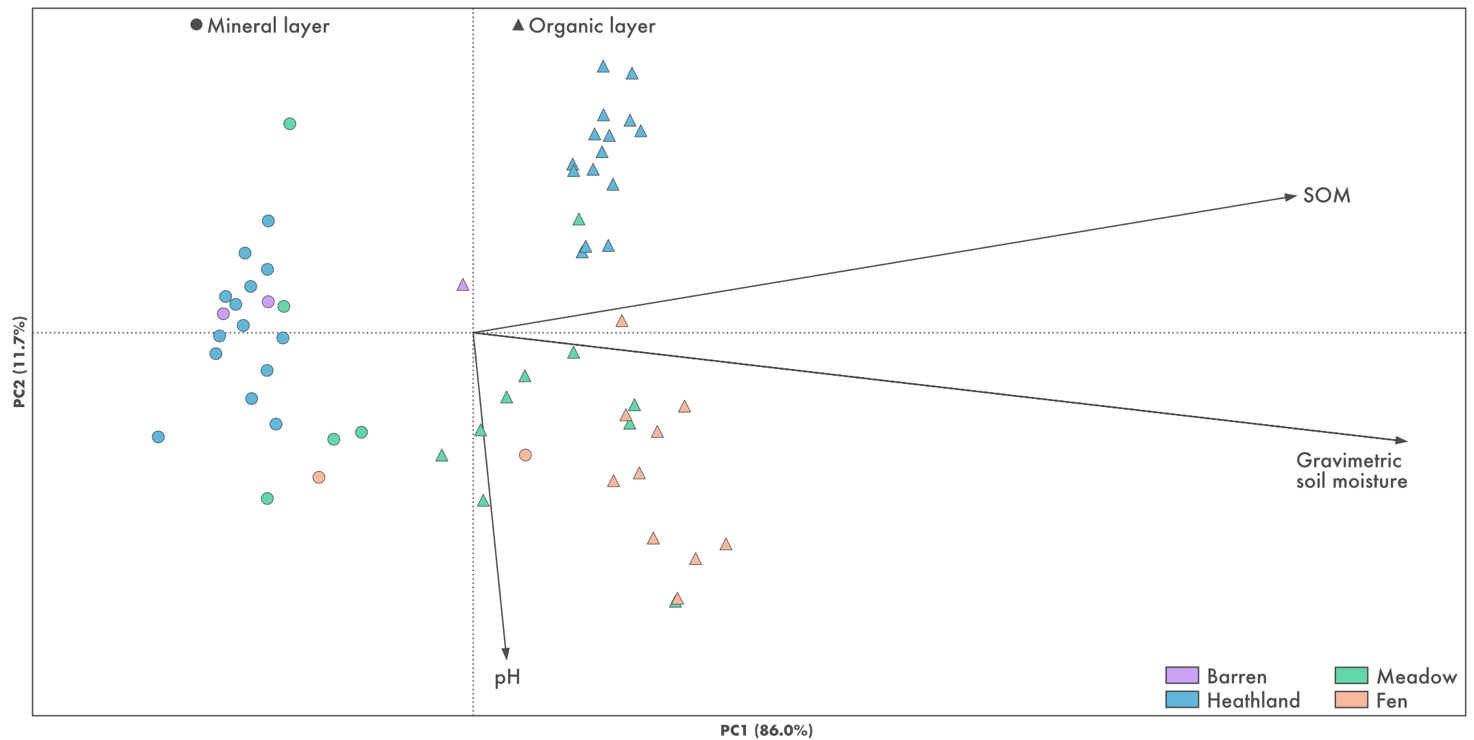


**Fig. S1. Physicochemical composition of tundra soils in Kilpisjärvi, northern Finland.** Principal component analysis (PCA) biplot showing differences in soil physicochemical composition – pH, gravimetric soil moisture and soil organic matter (SOM) – across different soil ecosystems and depths. More information about the samples can be found in **Additional file 1: Table S1**.


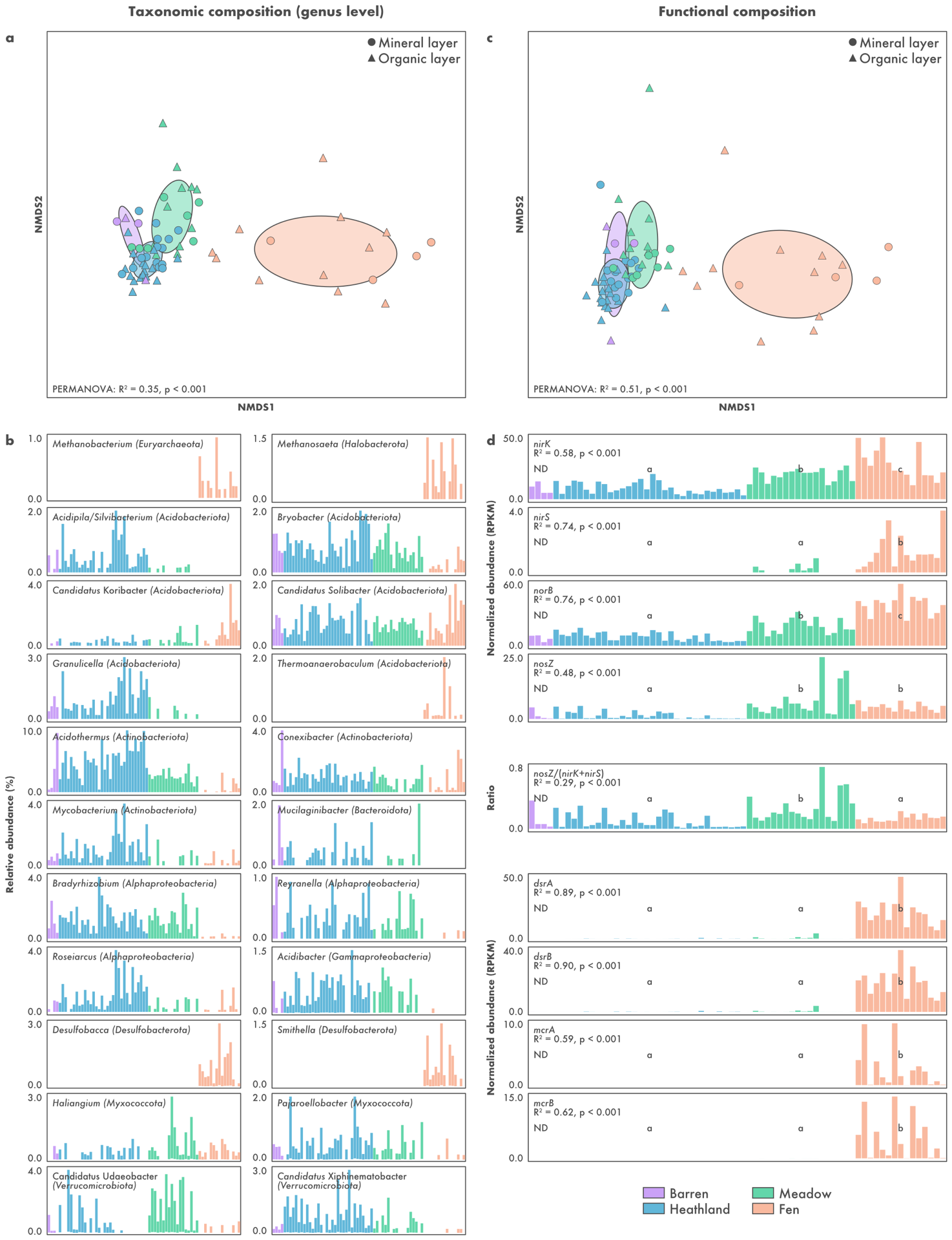


**(Previous page) Fig. S2. The microbial diversity of Kilpisjärvi soils as seen using a gene-centric approach.** Taxonomic composition was computed based on the annotation of unassembled SSU rRNA gene sequences against the SILVA database. Functional annotation was done by searching assembled genes against the KOfam database. The annotation of putative denitrification genes was confirmed using a three-step approach (see methods).
**a, c)** Non-metric multidimensional scaling (NMDS) of taxonomic and functional community structure, respectively. Differences between the ecosystems were assessed using permutational ANOVA (PERMANOVA). **b)** Abundance profile of the five most abundant genera in each ecosystem. **d)** Abundance profile of marker genes for denitrification, sulfate reduction, and methanogenesis. Ecosystems followed by different letters are significantly different (one-way ANOVA, p < 0.05). Samples from barren soils were not included in the ANOVA procedure due to the limited number of samples (ND: not determined).


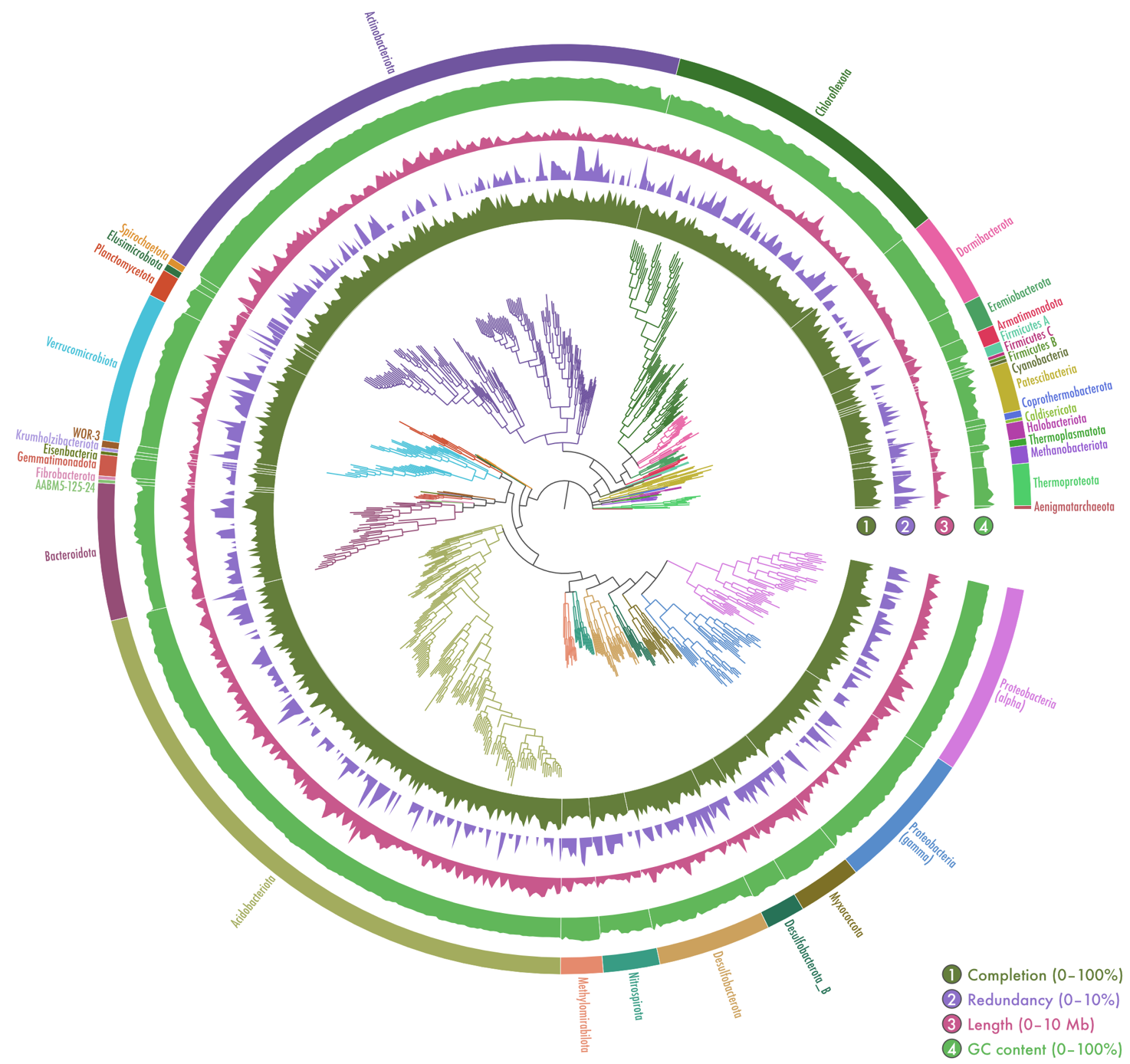


**Fig. S3. Genome-resolved metagenomics of tundra soils.** Phylogenomic placement and assembly statistics of 796 metagenome-assembled genomes (MAGs) recovered from soils in Kilpisjärvi, northern Finland. Unrooted maximum likelihood tree based on concatenated alignments of amino acid sequences from 122 archaeal and 120 bacterial single-copy genes. More information about the MAGs can be found in **Additional file 1: Table S2**.


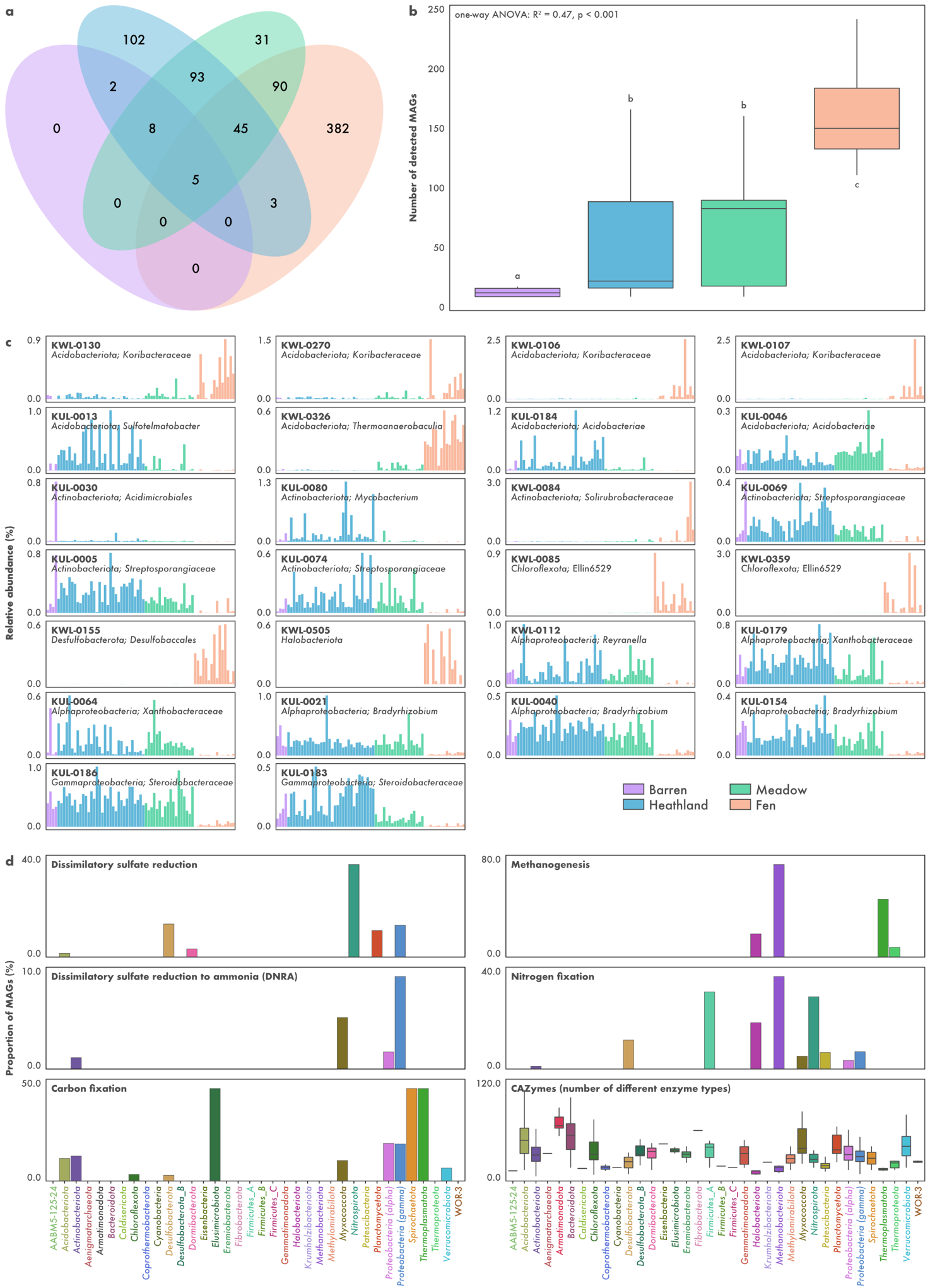


**(Previous page) Fig. S4. Overview of the microbial diversity in Kilpisjärvi soils based on a genome-resolved approach. a)** Number of metagenome-assembled genomes (MAGs) shared between the different ecosystems. **b)** Number of detected MAGs across the ecosystems. **c)** Relative abundance of the ten most abundant MAGs in each ecosystem, computed as a proportion of reads mapping to each MAG. **d)** Metabolic potential of the MAGs based on the annotation of genes against the KOfam database. Bar plots represent the proportion of MAGs in each phylum with complete pathways, i.e., containing ≥ 75% of the genes in the pathway. Boxplots of carbohydrate-active enzymes (CAZymes) show the number of different enzyme types identified in each MAG.


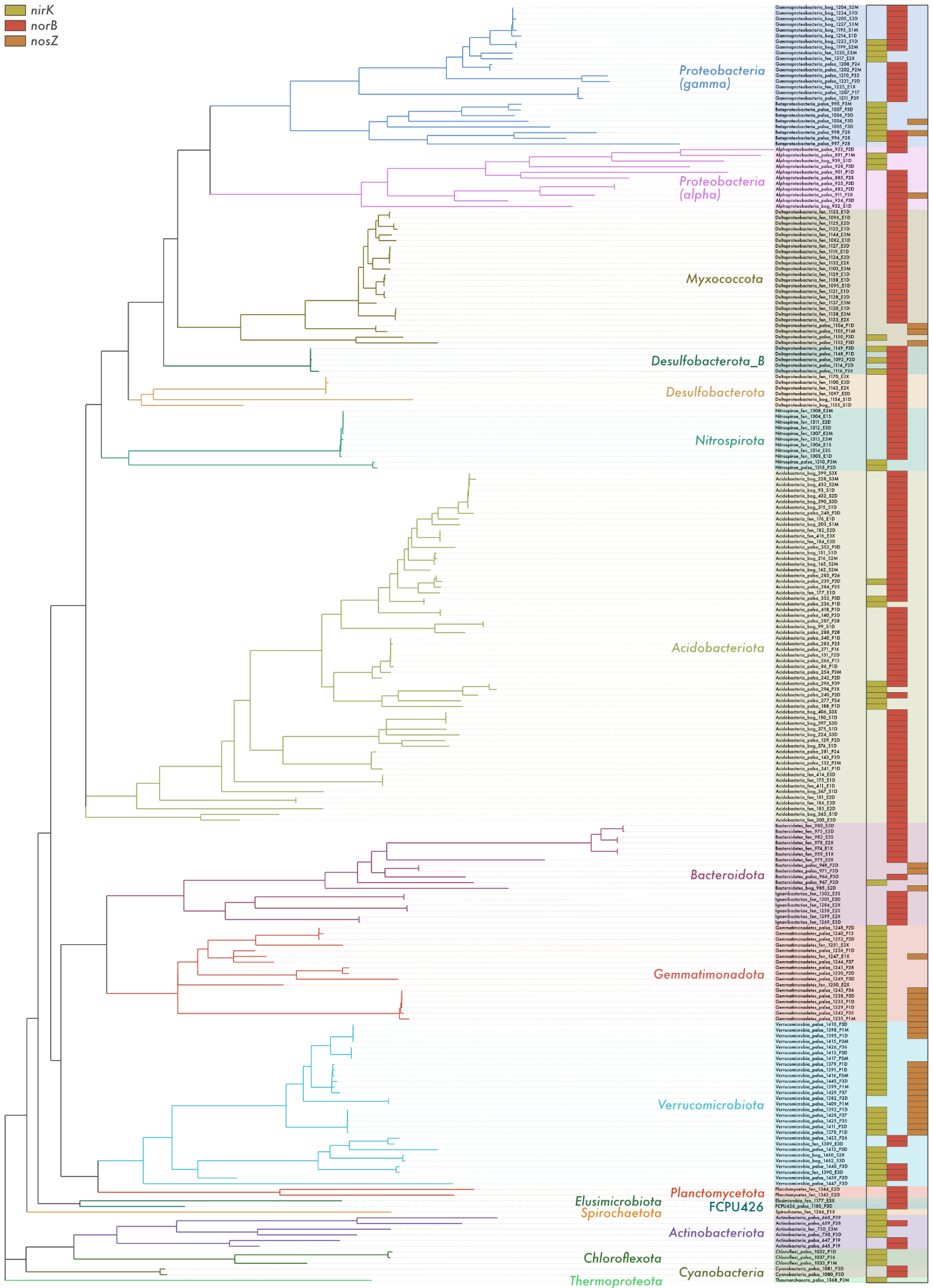


**(Previous page) Fig. S5. Metabolic potential for denitrification in Stordalen Mire soils.** Distribution of denitrification genes across 225 metagenome-assembled genomes (MAGs) from permafrost peatland, bog, and fen soils in Stordalen Mire, northern Sweden. Genes encoding the nitrite (*nirK*), nitric oxide (*norB*), and nitrous oxide (*nosZ*) reductases were annotated using a three-step approach (see methods). Phylogenomic analysis of MAGs was done based on concatenated alignments of amino acid sequences from 122 archaeal and 120 bacterial single-copy genes.


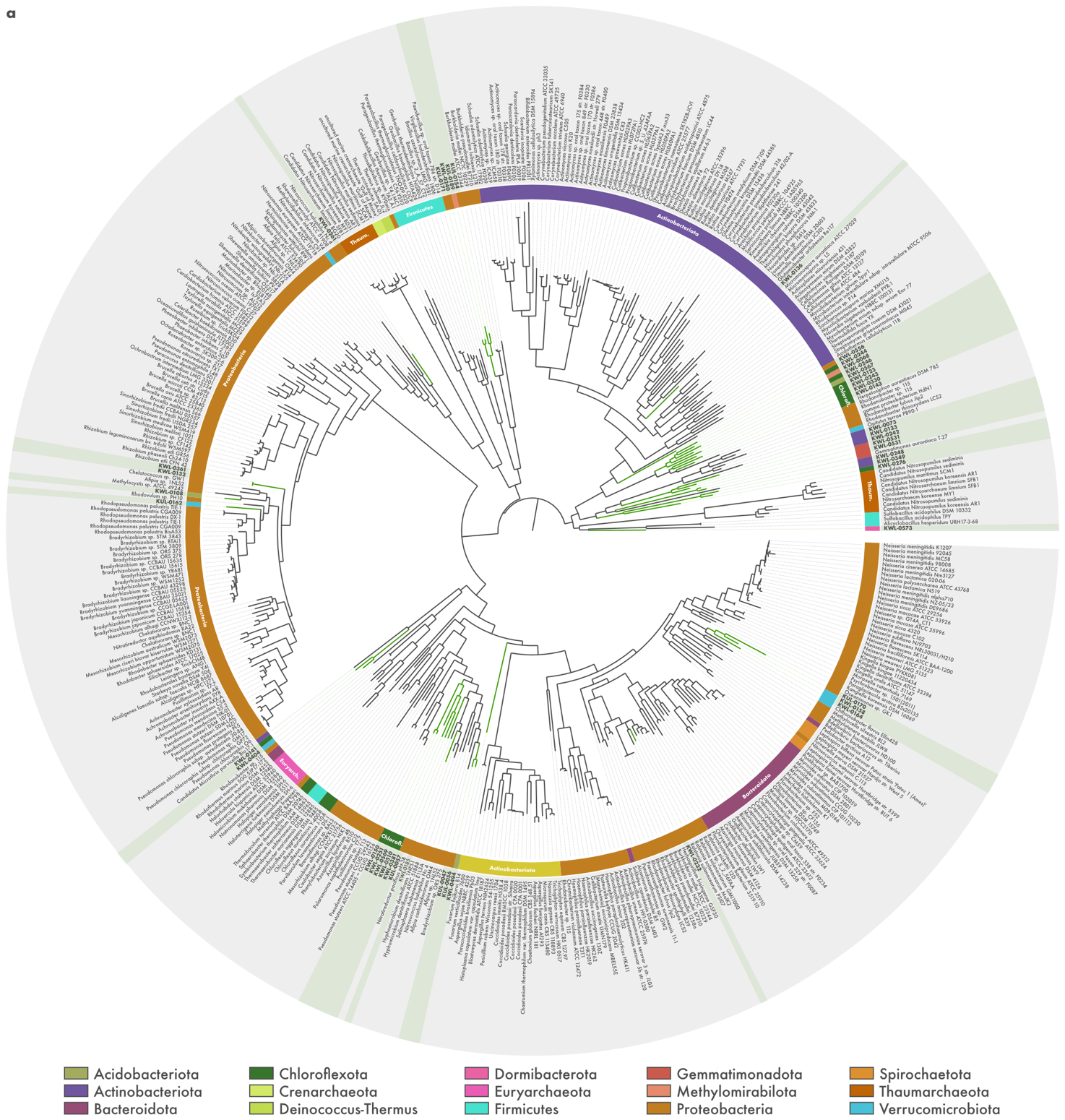


**Fig. S6. Phylogeny of a) *nirK*, b) *nirS*, c) *norB*, and d) *nosZ* sequences from metagenome-assembled genomes (MAGs) recovered from tundra soils in Kilpisjärvi, northern Finland.** Midpoint-rooted maximum-likelihood trees of translated sequences from Kilpisjärvi MAGs (highlighted) along with reference sequences from archaeal and bacterial genomes.


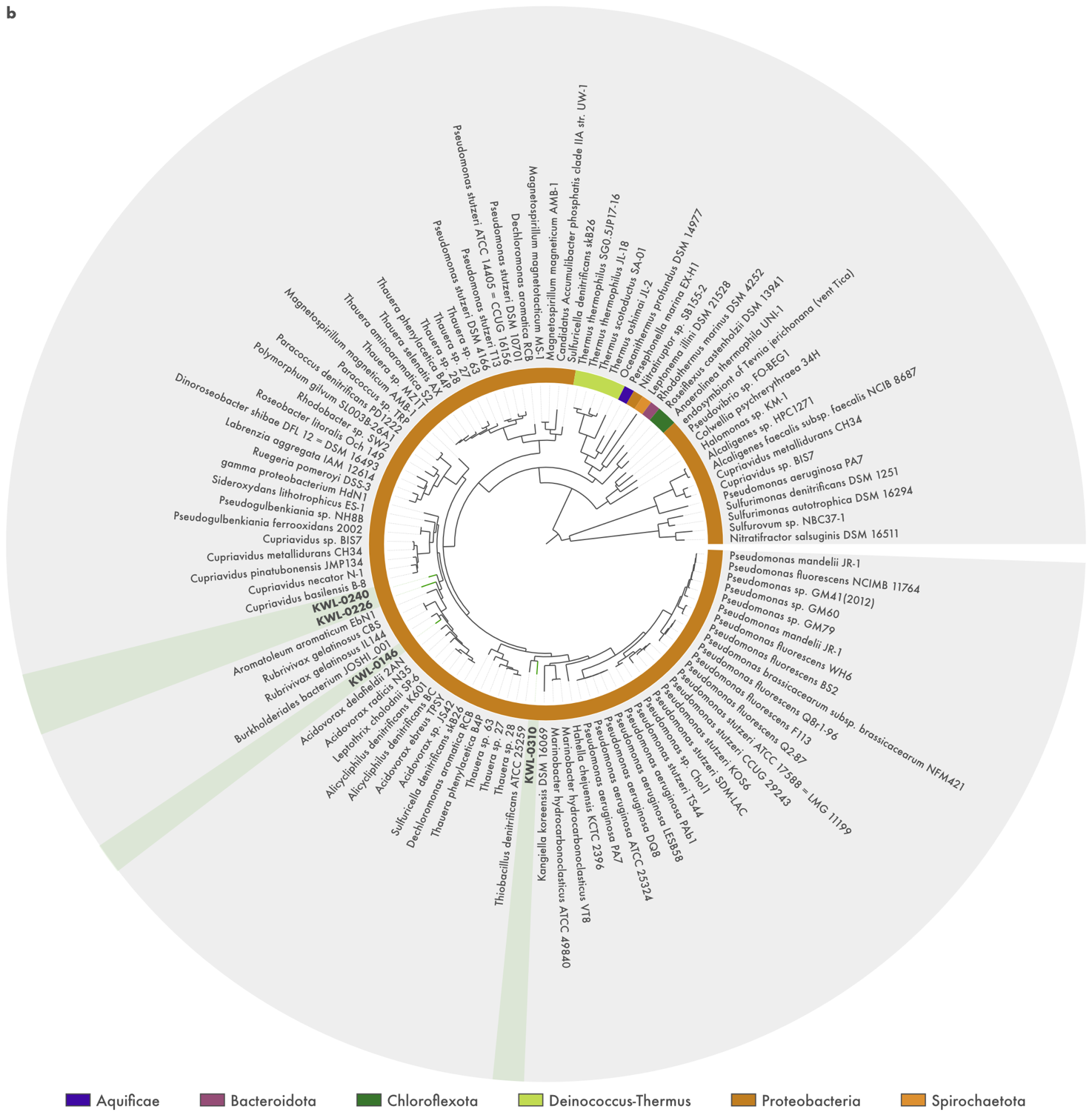


**Fig. S6 (continued). Phylogeny of a) *nirK*, b) *nirS*, c) *norB*, and d) *nosZ* sequences from metagenome-assembled genomes (MAGs) recovered from tundra soils in Kilpisjärvi, northern Finland.** Midpoint-rooted maximum-likelihood trees of translated sequences from Kilpisjärvi MAGs (highlighted) along with reference sequences from archaeal and bacterial genomes.


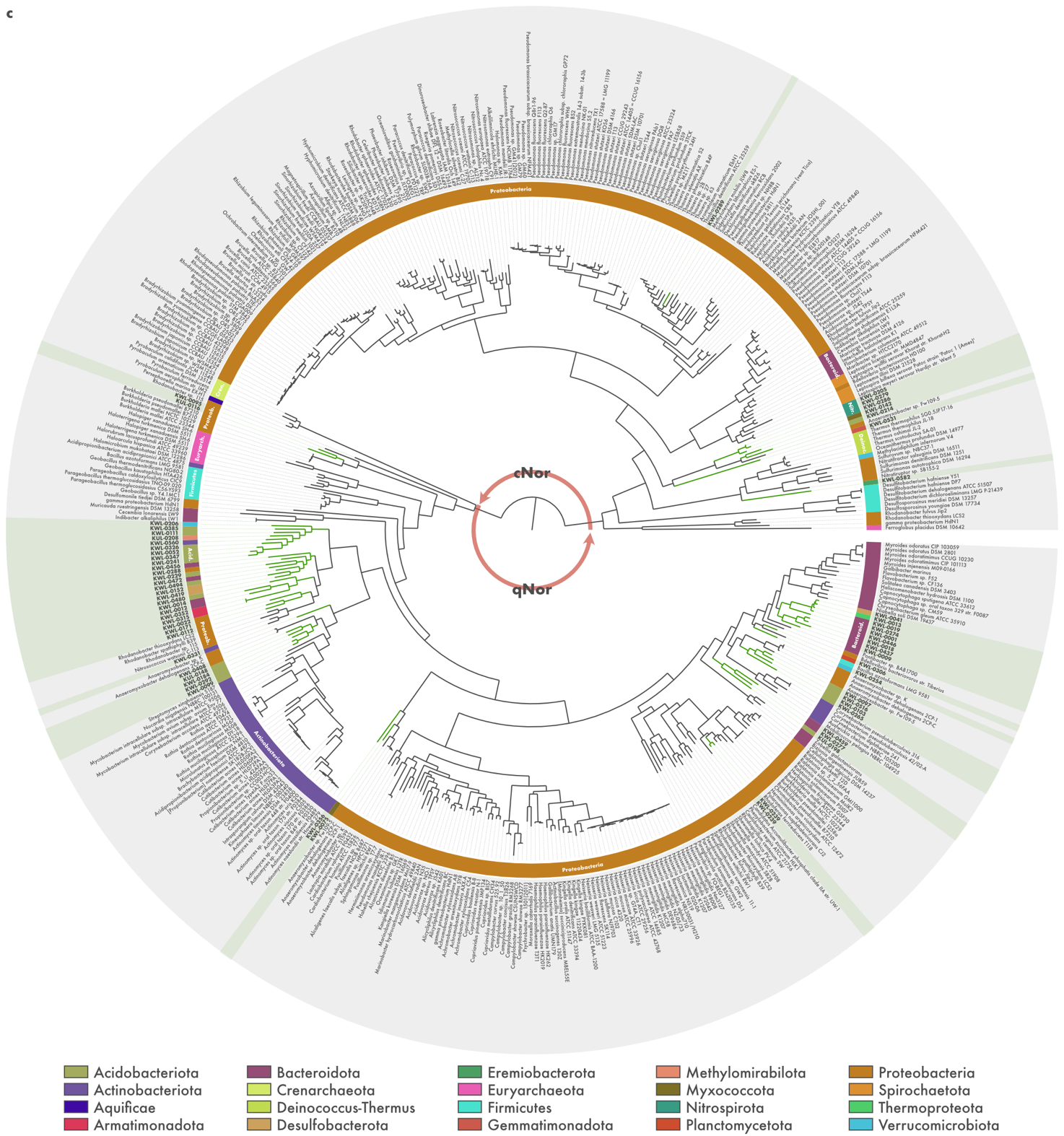


**Fig. S6 (continued). Phylogeny of a) *nirK*, b) *nirS*, c) *norB*, and d) *nosZ* sequences from metagenome-assembled genomes (MAGs) recovered from tundra soils in Kilpisjärvi, northern Finland.** Midpoint-rooted maximum-likelihood trees of translated sequences from Kilpisjärvi MAGs (highlighted) along with reference sequences from archaeal and bacterial genomes.


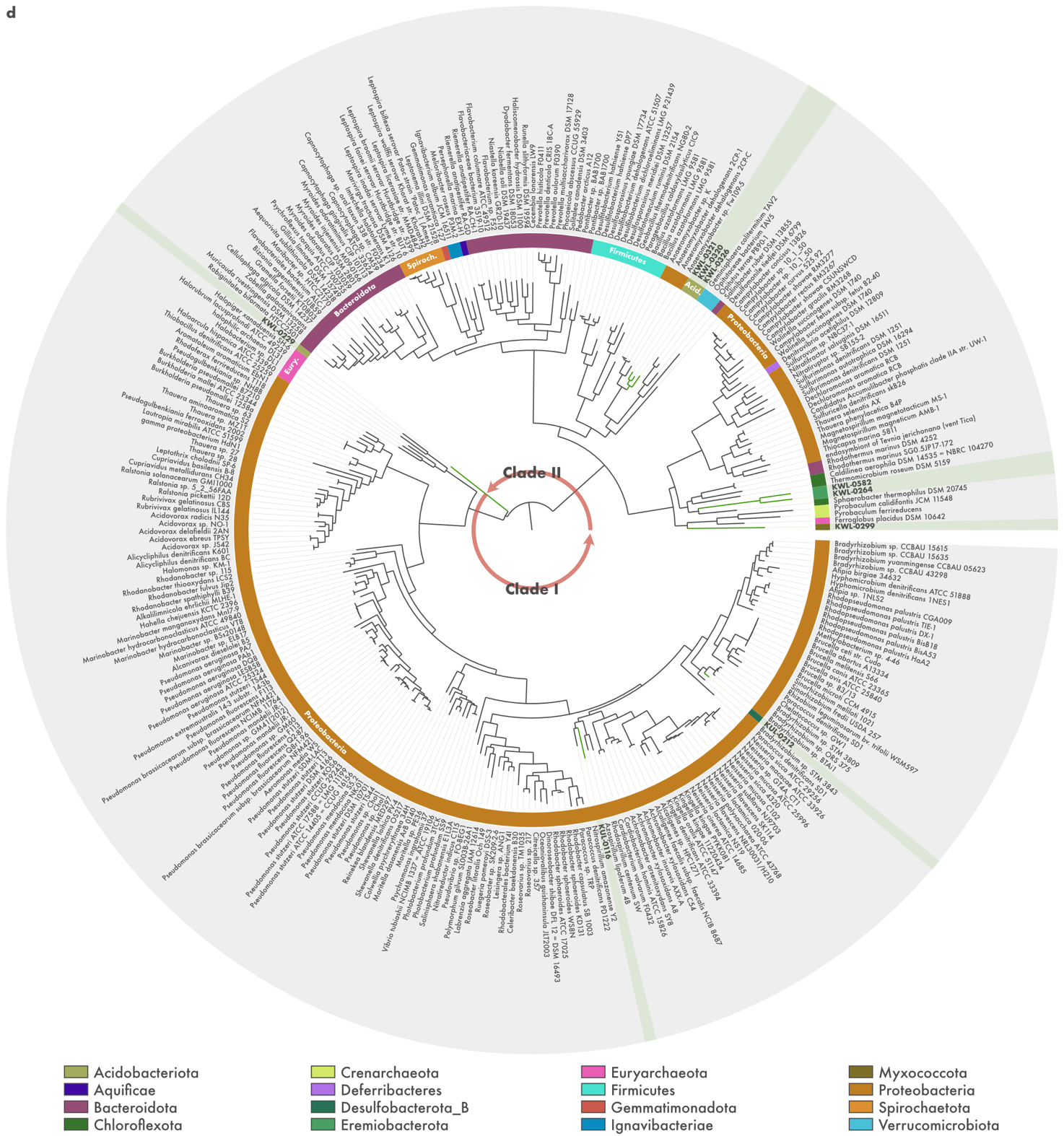


**Fig. S6 (continued). Phylogeny of a) *nirK*, b) *nirS*, c) *norB*, and d) *nosZ* sequences from metagenome-assembled genomes (MAGs) recovered from tundra soils in Kilpisjärvi, northern Finland.** Midpoint-rooted maximum-likelihood trees of translated sequences from Kilpisjärvi MAGs (highlighted) along with reference sequences from archaeal and bacterial genomes.
